# Clinical Utility of the Detection of the Loss of the Mismatched HLA in Relapsed Hematological Patients After Haploidentical Stem Cell Transplantation With High-Dose Cyclophosphamide

**DOI:** 10.3389/fimmu.2021.642087

**Published:** 2021-03-25

**Authors:** Paula Muñiz, Mi Kwon, Diego Carbonell, María Chicano, Rebeca Bailén, Gillen Oarbeascoa, Julia Suárez-González, Cristina Andrés-Zayas, Javier Menárguez, Nieves Dorado, Ignacio Gómez-Centurión, Javier Anguita, José Luis Díez-Martín, Carolina Martínez-Laperche, Ismael Buño

**Affiliations:** ^1^Department of Hematology, Gregorio Marañón General University Hospital, Madrid, Spain; ^2^Gregorio Marañón Health Research Institute (IiSGM), Madrid, Spain; ^3^Genomics Unit, Gregorio Marañón General University Hospital, Madrid, Spain; ^4^Pathology Department, Gregorio Marañón General University Hospital, Madrid, Spain; ^5^Department of Medicine, School of Medicine, Complutense University of Madrid, Madrid, Spain; ^6^Department of Cell Biology, School of Medicine, Complutense University of Madrid, Madrid, Spain

**Keywords:** HLA-loss, immune evasion, post-transplantation relapse, haploidentical stem cell transplantation, cyclophosphamide

## Abstract

Haploidentical hematopoietic stem cell transplantation (Haplo-HSCT) with high-dose cyclophosphamide (PTCy) has resulted in a low incidence of graft-vs.-host disease (GVHD), graft failure, and non-relapse mortality. However, post-transplantation relapse remains a common cause of treatment failure in high-risk patients. Unraveling the mechanisms of relapse is therefore crucial for designing effective relapse treatment strategies. One of these mechanisms is the loss of the mismatched HLA on the recipient's leukemic cells. To study the incidence and clinical relevance of this phenomenon, we analyzed 181 patients treated with Haplo-HSCT with PTCy (2007–2019), of which 37 relapsed patients after transplantation. According to the kit employed for HLA-loss analysis, among 22 relapsed patients, we identified HLA loss at relapse in 6 of the 22 patients (27%) studied. Based on the results obtained, the genomic loss of HLA was more common in females than males (66 vs. 33%) and HLA-loss relapses occurred later than classical relapses (345 vs. 166 days). Moreover, the patients with HLA-loss had a greater presence of active disease at the time of transplantation and had undergone a larger number of treatment lines than the group with classical relapses (66 vs. 43% and 66 vs. 18%, respectively). Four of these relapses were studied retrospectively, while two were studied prospectively, the results of which could be considered for patient management. Additionally, two relapsed patients analyzed retrospectively had myeloid neoplasms. One patient had not undergone any treatment, and three had undergone donor lymphocyte infusions (DLIs) and chemotherapy. All presented severe GVHD and disease progression. In contrast, the two patients studied prospectively had a lymphoid neoplasm and were not treated with DLIs. One of them was treated with chemotherapy but died from disease progression, and the other patient underwent a second Haplo-HSCT from a different donor and is still alive. We can conclude that the detection of HLA-loss at the onset of relapse after Haplo-HSCT with PTCy could help in clinical practice to select appropriate rescue treatment, thereby avoiding the use of DLIs or a second transplantation from the same donor.

## Introduction

Allogeneic hematopoietic stem cell transplantation (allo-HSCT) is the only curative option for many high-risk hematological malignancies (1). For many years, HLA-matched donors were the only type of donors employed. However, only 25% of patients who require an allo-HSCT have an HLA-identical sibling (2). Haploidentical HSCT (Haplo-HSCT) is therefore an interesting option for patients with an indication for transplantation (3). Several reports have shown comparable outcomes between Haplo-HSCT and a series of matched related donors, matched unrelated donors and mismatched unrelated donors (4–7). The success of the transplant lies in the donor immune system's capacity to remove residual leukemia cells via a graft-vs.-leukemia effect (GVL) based on the HLA disparity between donor and recipient, where donor T cells recognize patient-specific HLA molecules and eliminate leukemic cells. However, this effect is usually accompanied by an autoimmune and alloimmune disorder called graft-vs.-host disease (GVHD) (8). In this context, a variety of GVHD prophylaxis strategies have been published, one of which is the use of high-dose post-transplantation cyclophosphamide (PTCy), which eliminates expanding alloreactive T cells without affecting stem cells. This strategy would prevent the development of GVHD while maintaining immune reconstitution and thus the ability to control relapses (9–11). Haplo-HSCT can therefore achieve durable remissions and an acceptable incidence of GVHD. However, disease relapse is a significant obstacle to long-term survival (12). Considering that post-transplantation relapse is the most common cause of treatment failure, it is important to characterize the mechanisms of relapse after Haplo-HSCT. One of the results of the best-characterized tumor-intrinsic mechanisms of immune evasion and relapse is the “striking out” of interactions between T-cells and the tumor. This can occur when blast cells become “invisible” to T-cells, for instance due to alterations in the antigen processing and presenting machinery or because their interaction is inefficient, such as when inhibitory immune checkpoints are imposed (13). The genomic loss of HLA (copy neutral loss of heterozygosity), the epigenetic downregulation of class II HLA and the epigenetic upregulation of inhibitory molecules (*PDL1, B7H3, PVR* or *PVRL2*) are the three known tumor-intrinsic mechanisms (14).

Genomic HLA alterations have long been recognized in solid tumors. In hematological tumors, alterations in the HLA region are uncommon, especially at the time of diagnosis, a feature critical in Haplo-HSCT where donor T-cell-mediated alloreactivity converges against the recipient's incompatible HLA molecules that rapidly become the immunodominant GVL targets. However, various studies have shown that class I and II HLA-loss in the leukemic blasts at relapse, in particular from the HLA allele mismatched between donor and recipient, can occur in 30% of acute myeloid leukemia relapses after Haplo-HSCT (15, 16). A deeper examination of this phenomenon has demonstrated a loss of heterozygosity (LOH) of chromosome 6p in the absence of copy number variations, thus suggesting an event of acquired somatic uniparental disomy (aUPD). In other words, LOH might not be due to a loss of chromosomal material. aUPD consists of the loss of a region of the chromosome and the replacement by the exact copy of the homologous chromosome (either paternal or maternal), resulting in acquired homozygosity of that region without the loss of genomic material (13).

At the time of relapse, various rescue treatments can be employed, such as withdrawing immunosuppressive drugs and infusing donor lymphocytes (DLIs) (to improve the GVL effect) (12, 17) and chemotherapy (to achieve complete remission as a “bridge” prior to a second transplantation) (12, 18). Nevertheless, in HLA-loss relapses, attempting to improve GVL through DLIs would not be an optimal strategy, given that leukemic cells become invisible to donor T cells (13). Furthermore, such approaches can cause severe complications such as GVHD (19). Better options would be to employ chemotherapy, hypomethylating agents, therapeutic targets and other novel drugs for patients who present HLA-loss. Considering this genomic alteration, another useful strategy could be a second Haplo-HSCT from an alternative donor who shares the other HLA haplotype with the recipient (20). In this context, donor T cells will still share a haplotype with non-hematopoietic tissues from the patient but a 100% HLA mismatch with the relapsed leukemia, thus providing an even stronger GVL effect (21). A different haploidentical donor will carry T-cells that are alloreactive against the HLA haplotype conserved by the leukemic blasts. In the series of HLA-loss relapses reported by Crucitti et al. (16), a second transplant from an alternative donor was the treatment associated with the longest overall survival (OS) (16).

While HLA-loss has been documented after Haplo-HSCT with methotrexate and antithymocyte globulin (15, 16), there have been few reports on HLA-loss after Haplo-HSCT followed by PTCy (22–24).

For the aforementioned reasons, understanding the mechanisms of immune-evasion leading to disease relapse will help in selecting the most effective rescue treatment. In this context, the aim of this study was to analyze HLA-loss relapses, as a mechanism of leukemia immune escape, in a large cohort of hematological patients who relapsed after T-cell replete Haplo-HSCT with PTCy.

## Materials and Methods

### Patients

We retrospectively selected 181 consecutive patients with hematological malignancies who underwent Haplo-HSCT with PTCy at Gregorio Marañón General University Hospital (Madrid, Spain) from December 2007 to June 2019. Of the entire cohort, 37 patients relapsed. We collected data until March 2020. The local ethics committee approved the study, and all recipients and donors provided written informed consent according to the Declaration of Helsinki.

The conditioning regimen for Haplo-HSCT was myeloablative for 78 patients and reduced intensity conditioning for 103 patients. The myeloablative conditioning regimen consisted of fludarabine 40 mg/m^2^/day from day −6 to day −3 and intravenous busulfan 3.2 mg/kg/day on either day −6 to −3. The reduced intensity conditioning regimen included fludarabine 30 mg/m^2^/day on day −6 to day −2, intravenous busulfan 3.2 mg/kg/day on day −3, and cyclophosphamide (14.5 mg/kg) on day −6 and day −5. Prophylaxis against GVHD consisted of high-dose PTCy (50 mg/kg) administered on day +3 and day +4 post-transplantation, followed by tacrolimus and mycophenolate mofetil 10 mg/kg/8h from day +5. In the absence of GVHD, mycophenolate mofetil was discontinued on day +35.

### Methods

Genomic DNA was extracted from bone marrow (BM) or peripheral blood (PB), upon diagnosis and during the post-transplantation follow-up, according to the manufacturer's instructions using a Maxwell® RSC Blood DNA Kit (Promega, USA). Specifically for lymphoproliferative syndrome (LPS) without BM infiltration, tumor DNA was obtained from formalin-fixed paraffin-embedded (FFPE) affected tissues using a Maxwell® RSC DNA FFPE Kit (Promega, USA). Post-transplantation hematopoietic chimerism was evaluated using short tandem repeat (STR) PCR amplification (*AmpFlSTR SGM Plus, Thermo Fisher Scientific, MA, USA*) with a sensitivity of 1% (25) (Other methods have not been employed for chimerism study). A chimerism analysis was conducted on day +15, day +30 and monthly thereafter for acute leukemias and LPS with BM infiltration. In contrast, in LPS without BM involvement, the chimerism analysis was conducted until complete chimerism was achieved.

#### Loss of Mismatched HLA Studies

Pre-HSCT HLA compatibility among the recipients and donors was analyzed by the Transfusion Center of Madrid through both a sequence-specific oligonucleotide and sequence-based typing polymerase chain reaction. The loci included in the pretransplantation HLA compatibility study were HLA class I (HLA-A, HLA-B and HLA-C) and HLA class II (HLA-DR and HLA-DQ). Given that the DPB1 locus was not analyzed, relapses in which this allele was involved were not considered in the current study.

The HLA-loss study in relapsed patients is carried out once active disease has been confirmed through flow cytometric and genetic analysis in myeloid neoplasms and immunohistochemical and genetic analysis for lymphoproliferative disorders.

In the relapsed patients, the recipient-specific HLA was selected according to the pre-HSCT HLA compatibility study among recipients and donors. The study of recipient-specific HLA in the relapsed patients was performed by real-time quantitative PCR using the HLA-KMR kit (GenDX, Netherlands) (26) with a LightCycler 480 System (Roche, Switzerland) according to the manufacturer's protocol. The relapse study was performed from FFPE samples for patients 4,5,9 and 20. The kit enables testing for HLA-A, -C, and -DPB1 alleles (which were not considered in the present study).

The chimerism analysis and loss of mismatched HLA studies were performed on the same relapse DNA sample.

This PCR approach provides univocal discrimination of HLA loss from classical relapses. However, caution is warranted when there are suspected non-tumoral cells in the recipient, because this could lead to false negative results, which can happen especially in FFPE samples. For this reason, an internal control (included in the employed kit) was included for the HLA-loss studies with FFPE samples to check the sample's quality. The analysis of the results should be performed with caution. If there is amplification of the HLA markers, the analysis cannot ensure that there has been no HLA-loss, given that stromal elements might have interfered with the results. On the other hand, when there is no HLA marker amplification, the results indicate that the FFPE sample has had HLA loss at relapse.

#### Statistical Analysis

The quantitative variables are expressed as medians and ranges, while the categorical variables are expressed as frequencies and percentages. We employed Fisher's exact test to compare the distribution of the categorical variables and the Mann-Whitney test to compare the differences between two independent variables. We calculated the cumulative incidence of relapse using the Fine-Gray test from the time complete remission was achieved until the date of the event (in case of relapse) of the last examination (for those patients who did not undergo an event) or of the emergence of the competing risk (for those patients who died or underwent transplantation without a subsequent event). We calculated the overall survival (OS) from the occurrence of post-transplantation relapse using the Kaplan-Meier method, censoring patients at the time of death or last follow-up.

We performed the statistical analysis using SPSS v.26 (IBM Corporation, USA), R version 3.5.1 and the “cmprsk” package.

#### Definitions

We defined classical relapses as those in which the recipient-specific HLA alleles remained at relapse. Conversely, we defined HLA-loss relapses as those in which the recipient tumor DNA showed genomic loss of the donor-recipient mismatched HLA.

## Results

Of the 181 patients who underwent Haplo-HSCT, 37 patients relapsed with a cumulative incidence of relapse of 19.5% at 3 years. In terms of the HLA-loss studies and considering the HLA-A and HLA-C alleles, we were able to study 22 (59.5%) of the relapsed cases. We excluded 15 patients from the analysis because the kit employed did not include the recipient-specific HLA alleles. We therefore analyzed a total of 22 relapse cases (Tables 1, 2 and Supplementary Table 1). Four cases were studied prospectively, and the remaining patients were studied retrospectively (Supplementary Table 2).

**Table 1:** Chimerism and relapse data of 22 patients relapsed after Haplo-HSCT with PTCy.

**Patient**	**Diagnosis**	**Chimerism at HLA-loss study (% Recipient)^†^**	**Sample type**	**Relapse details (MRD (Flow cytometry and genetic) and imaging evaluation)**			**HLA-loss relapse**	**Exitus**
1	DCL	5	BM	30% blasts	MRD +		Yes	Yes
2	AML	1.6	BM	CNS infiltration	MRD +	WT1 +	Yes	Yes
3	MDS	1.5	BM	7% blasts	WT1 +		Yes	Yes
4	HL	26	Pelvic adenopathy	Mediastinal adenopathy	PET/CT positive		Yes	Yes
5	HL	13.6	Axillary adenopathy	Supra/infradiaphragmatic relapse	PET/CT positive		Yes	No
6	ALL-T	11	PB	17% blasts	MRD +		Yes	Yes
7	AML	23	PB	24% blasts	MRD +	WT1 +	No	Yes
8	NHL	2.5	BM	BM infiltration 25% blasts. Axillary and mediastinal adenophaty	MRD +	PET/CT positive	No	Yes
9	NHL	38	Hepatic adenopathy	Liver infiltration	PET/CT positive		No	Yes
10	ALL-B	7.8	PB	60% blasts	MRD +		No	Yes
11	NHL	44	BM	CNS + BM infiltration 25% blasts	MRD +		No	Yes
12	AML	12	PB	28% blasts	MRD +	FLT3 /WT1 +	No	No
13	AML	88	PB	90% blasts	MRD +	NPM1/WT1 +	No	Yes
14	ALL-B	24	BM	Mediastinal adenopathy. CNS + BM 5% infiltration	MRD +	PET/CT positive	No	Yes
15	ALL-B	1.5	PB	62% blasts	MRD +		No	Yes
16	AML	1.6	BM	10% blasts	MRD +	WT1 +	No	No
17	AML	31	BM	46% blasts	MRD +	WT1 +	No	Yes
18	AML	20.6	BM	25% blasts	MRD +	WT1 +	No	Yes
19	AML secondary to NHL	97	PB	90% blasts	MRD +	WT1 +	No	Yes
20	HL	22	Cervical adenopathy	Cervical adenopathy	PET/CT positive		No	Yes
21	AML	76	PB	Medullary aplasia	FLT3/NPM1 +		No	Yes
22	AML	90	BM	86% blasts	MRD +		No	Yes

ALL-B, B-cell acute lymphoblastic leukemia; ALL-T, T-cell acute lymphoblastic leukemia; AML, acute myeloid leukemia; CNS, central nervous system; CSF, cerebrospinal fluid; DCL, dendritic cell leukemia; GVHD, graft-vs.-host disease; HL, hodgkin's lymphoma; HSCT, hematopoietic stem cell transplantation; MRD, minimal residual disease; MDS, myelodysplastic syndrome; NHL, non-hodgkin's lymphoma. MRD +, MRD positive; WT1+, overexpression WT1.

† *Post-transplantation hematopoietic chimerism was evaluated using short tandem repeat. PET/CT was employed for study relapsed patients with lymphoproliferative syndrome without bone marrow infiltration*.

**Table 2:** Clinical characteristics of 22 patients relapsed after Haplo-HSCT with PTCy.

			**HLA-loss relapses (*n* = 6)**	**%**	**Classical relapses (*n* = 16)**	**%**	***P*-value**
Diagnosis	AML		1	16	9	56	0.64
	MDS		1	16			
	DCL		1	16			
	ALL-B				3	18	
	ALL-T		1	16			
	HL		2	33	1	6	
	NHL				3	18	
Age (years) (Median, range)			45 (27–55)		31 (17–65)		0.28
Recipient Sex (Female/Male)			4/2	66/33	6/10	37/62	0.35
Donor Sex (Female/Male)			3/3	50/50	11/5	68/31	0.62
Previous HSCT	Allogeneic		2	33	2	12	0.63
	Autologous		1	16	2	12	
Treatment lines pre-HSCT	≤2		2	33	13	81	0.06
	>2		4	66	3	18	
Status at transplant	Active disease		1	16	3	18	0.89
	Partial Response		3	50	4	25	
	Complete Remission		2	33	9	56	
Conditioning regimen	Myeloablative		3	50	9	56	0.92
	Non-Myeloablative		3	50	7	43	
Time from HSCT to relapse (days) (Median, range)			345 (88–570)		166 (48–1458)		0.15
GVHD before relapse	Acute	Grade I	2	33	3	18	0.35
		Grade II	1	16	3	18	
		Grade III	1	16	2	12	
	Chronic	Mild	1	16	2	12	0.58
		Moderate	-	-	-	-	
		Severe	-	-	1	6	

*ALL-B, B-cell acute lymphoblastic leukemia; ALL-T, T-cell acute lymphoblastic leukemia; AML, acute myeloid leukemia; DCL, dendritic cell leukemia; GVHD, graft-vs.-host disease; HL, hodgkin's lymphoma; HSCT, hematopoietic stem cell transplantation; MDS, myelodysplastic syndrome; NHL, non-hodgkin's lymphoma*.

In our cohort, genomic loss of the recipient-specific HLA occurred in 6/22 patients (27%) (Table 2). These patients presented various hematological neoplasms: 1 acute myeloid leukemia, 1 myelodysplastic syndrome, 4 lymphoid neoplasms (1 T-cell acute lymphoblastic leukemia, 1 dendritic cell leukemia and 2 Hodgkin's lymphomas). The genomic loss of HLA was more common in females than in males (66.6 vs. 33.3%, not statistically significant [NS]) and the HLA-loss relapses occurred later after HSCT than the classical relapses (median 345 vs. 166 days, NS) (Table 2). Moreover, the proportion of patients with active disease at the time of transplantation and the number of treatment lines prior to transplantation were higher in the patients with HLA-loss than in those with classical relapse (respectively 66 vs. 43%, NS; 66 vs. 18%, NS).

**Figure 1:**
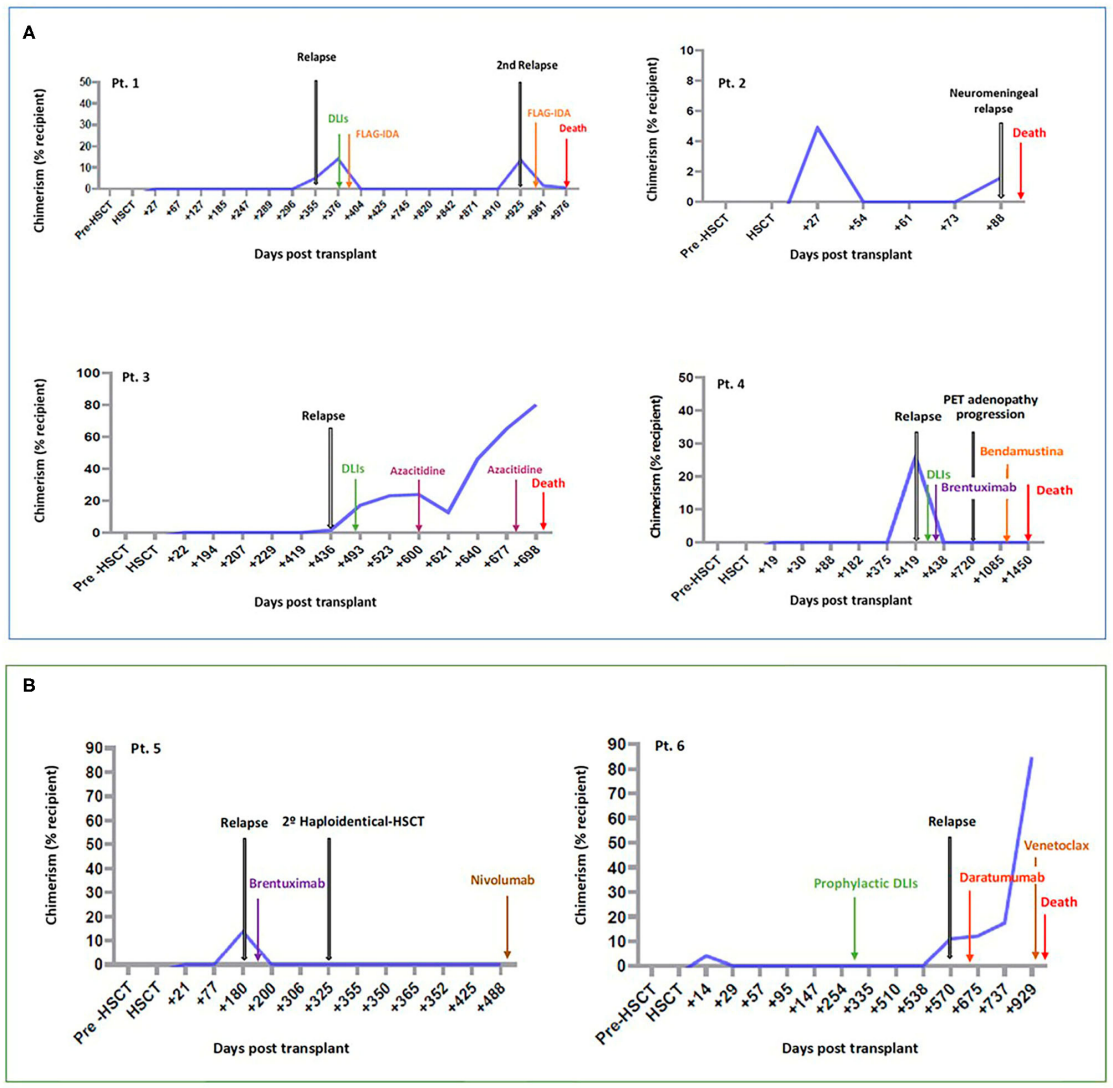
Patients with HLA-loss at relapse. Follow-up of patients in which HLA-loss was identified retrospectively **(A)** and patients identified prospectively, in which HLA-loss could be used to drive therapeutic decisions **(B)**. Pt, Patient; HSCT, hematopoietic stem cell transplantation; DLIs, Donor lymphocyte infusions.

None of the patients with HLA-loss relapse were undergoing immunosuppressive therapy at the time of the relapse. Patients 1-4 were analyzed retrospectively (Figure 1A), three of whom had been administered DLIs plus chemotherapy or other drugs for treating the relapse. These three patients treated with DLIs developed severe post-treatment GVHD (patient 1 developed grade II aGVHD, and mild cGVHD and patients 3 and 4 developed grade III-IV aGVHD) (Table 3 and Supplementary Table 2). Patient 2 did not undergo any treatment due to a neuromeningeal relapse (Supplementary Table 2). All patients died due to disease progression. In this patient subgroup, the OS at 6 and 12 months was 50 and 25%, respectively (Supplementary Figure 1).

**Table 3:** Rescue treatment and clinical outcome of patients with HLA-loss and classical relapses of post-transplantation.

		**HLA-loss relapse**		**Classical relapse**
		**Retrospective**	**Prospective**	
Number of patients (*n*)		4	2	16
Patient ID#		1–4	5,6	7–22
Treatment at relapse	No treatment	1	-	2
	Chemotherapy	-	2	9
	DLIs	-	-	1
	DLIs plus chemotherapy	3	-	4
Severe GVHD after rescue treatment		3	-	3
OS	At 6 months	50%	100%	31%
	At 1 year	25%	50%	18%

*Detailed information on the rescue treatments used and on the clinical outcome is provided in Supplementary Table 2. Severe GVHD includes III-IV aGVHD and cGVHD. DLIs, donor lymphocyte infusions; GVHD, graft-vs.-host disease; OS, overall survival*.

Prospectively, we detected HLA-loss relapse in 2 patients (patients 5 and 6) (Figure 1B, Table 3, and Supplementary Table 2). For the treatment of the relapse, patient 5 was administered brentuximab, achieved a partial response and subsequently underwent a second Haplo-HSCT from an alternative donor, achieving complete remission for 18 months. However, the patient presented a second relapse and is currently being treated with nivolumab. Lastly, patient 6 also relapsed, despite having been administered a dose of prophylactic DLIs. In this case, the relapse was treated with daratumumab with no response (17% blasts in BM and 7% in PB) and subsequently with venetoclax, but the patient died due to disease progression 1 year later. Neither patient 5 nor patient 6 developed GVHD after the rescue treatment. In this subgroup of patients, the OS at 6 and 12 months after relapse was 100 and 50%, respectively (Supplementary Figure 1).

The remaining 16 patients had classical (non-HLA-loss) relapses (Table 3 and Supplementary Table 2). The relapse treatment included immunosuppression tapering in patients 9, 10, 14, 15, and 21. Two patients did not undergo treatment (patients 13 and 19), six patients were treated with DLIs plus intensive treatment, and 8 patients underwent various intensive treatment approaches depending on the disease. Regarding the treatment with DLIs, two patients developed GVHD (patient 16 developed mild cGVHD, and patient 17 developed grade III-IV aGVHD). Specifically, patients 12, 18, and 22 were candidates for a second Haplo-HSCT from an alternative donor. Despite the relapse treatment, 14/16 (87.5%) patients died, 8 from disease progression, four from infectious complications and two who did not undergo any treatment. The OS at 6 and 12 months after relapse was 31 and 18%, respectively.

Taking into account the patient subgroup treated with DLIs at relapse after post Haplo-HSCT, the median survival was 375 days for the patients with HLA loss and 750 days for those with classical relapses (NS). However, the median survival for the patients who were not treated with DLIs at relapse was 375 days for the patients with HLA-loss and 125 days for those with classical relapses (NS).

Considering all patients with HLA-loss and classical relapses, the median survival time after relapse was 375 days for the patients with HLA-loss and 120 days for those with classical relapses. The outcomes of the patients with HLA-loss and those of the patients with classical relapses did not differ significantly (*p* = 0.4) (Supplementary Figure 1).

## Discussion

Relapse remains the major cause of mortality among patients who undergo Haplo-HSCT. The therapeutic options for patients with hematological neoplasms who relapse after Haplo-HSCT include palliative care, low-dose or intensive chemotherapy, cell therapies such as DLIs, and a second allo-HSCT in selected cases. Due to the low response rates and substantial toxicity, however, long-term survival is rare following these treatment modalities. A deeper understanding of the mechanisms by which patients relapse could therefore help in selecting the best treatment, increasing its effectiveness and reducing the toxicity of such treatment and thereby offering a personalized medical approach. One of the immune evasion mechanisms of leukemic cells to escape donor T-cell recognition is the loss of the patient-specific class I and II HLA antigens. In this study, we have examined the largest cohort of patients with HLA-loss at relapse after Haplo-HSCT with PTCy and we described HLA-loss for lymphoid neoplasms, which to our knowledge has not been reported to date. PTCy is a GVHD prophylaxis platform that induces the clonal destruction of alloreactive T cells. Indeed, hematopoietic stem cells from Haplo-HSCT have high levels of the cellular aldehyde dehydrogenase (ALDH), an enzyme that represents the major mechanism of cyclophosphamide detoxification given that hematopoietic stem cells that express high ALDH1A1 levels, they are relatively resistant to the effects of cyclophosphamide (27). For this reason, lymphocytes responsible for the GVL effect are not eliminated. Based on our results, we can therefore suggest that GVL with PTCy is not impaired, as a number of authors have previously shown (28). However, this immune pressure can lead to tumor cells losing the HLA haplotype, turning invisible to donor T cells and re-emerging. We showed in detail the HLA-loss in 22 patients who relapsed after Haplo-HSCT with PTCy.

There are currently various techniques for detecting HLA-loss. The use of chromosomal microarray analysis platforms combine classic copy number analysis with single-nucleotide polymorphism recognized as the first-line test for copy number variation detection. However, the drawbacks of this approach are its low sensitivity, given that LOH cannot be detected in lower level clones (<10%), and the moderate-to-high cost per sample. Another option is next-generation sequencing (NGS) based on the deep-sequencing of HLA genes, an approach that can also be employed to provide information at the allelic level. In a recent study, Vago et al. (29) reported on the NGS analysis of relapses after Haplo-HSCT (mean coverage > 8,500x). Chromosomal microarray analysis and NGS are cumbersome techniques, require several laborious steps to obtain results, require bioinformatic software to analyze the data, and present a high cost per sample. In contrast, real-time quantitative PCR (qPCR) is a reliable, sensitive and inexpensive assay to detect HLA-loss relapses, and results can be obtained the same day. To detect HLA-loss relapses in clinical practice, a fast, reliable, sensitive and cost-effective assay is required. The HLA-KMR qPCR kit developed by Ahci et al. (26) has a number of advantages, although it can be used to study only 70% of relapses because it only contains qPCR assays for HLA-A, HLA-C and HLA-DPB1 alleles. In this context and for the aforementioned reasons, qPCR approaches (such as that provided by the HLA-KMR kit) are an excellent option. The technique has high sensitivity, which enabled us to detect HLA-loss in the patients with incipient mixed chimerism (such as patients 2 and 3 with only 1% recipient cells), enabling early therapeutic decision making at relapse. However, we were unable to study all relapsed cases according to the available assays in the kit, due to the fact that the DPB1 allele, which is included in the kit, is usually not considered in the HLA compatibility study pre-HSCT. To get the most out of the kit and to increase the number of patients eligible for analysis, the DPB1 allele should be included in HLA compatibility studies. Another option could be to increase the number of markers for A and C alleles and design reagents to study the B alleles. To overcome these limitations, the most feasible alternative at present would be to sequence the HLA genes by NGS.

In this study, the incidence of HLA-loss was 27%, which is consistent with previous studies (20–40%) (15, 16).

Interestingly, most cases of HLA-loss relapses have been reported in patients with myeloid neoplasms (15, 16). In contrast, only 2/6 patients (33%) in our cohort with HLA-loss presented a myeloid neoplasm, while 4/6 patients were diagnosed with lymphoid neoplasms. To our knowledge, HLA-loss has not been reported after Haplo-HSCT for lymphoid neoplasms. There is only one report of HLA-loss in acute lymphoblastic leukemia after allo-HSCT, but in this study the relapse occurred after HSCT from related donors with HLA-DRB1 and HLA–DQB1 mismatches (30). The reported lack of HLA-loss relapses after LPS might be due to the difficulty in studying the relapse, because FFPE samples are not always accessible. We were able to conduct chimerism and HLA-loss studies in FFPE samples obtained from the pathology department, which highlights the advantages of performing patient care in multidisciplinary teams. These results suggest that HLA-loss is an immune evasion mechanism specific not only to myeloid neoplasms and that post-transplantation follow-up should be conducted for all types of hematological neoplasms and on different samples (BM, PB, and FFPE).

Crucitti et al. (16) showed that active disease pre-transplantation, a high number of treatment lines, younger patient age and chronic GVHD (cGVHD) could be risk factors for HLA-loss relapses (16). In our study, patients with active disease at the time of transplantation showed a higher frequency of HLA-loss than in the classical relapses (66 vs. 43%) but the difference did not reach statistical significance (Table 1). Although the numbers are small and only 2/6 patients with HLA-loss relapses were in complete remission pre-transplantation, our results are consistent with those of the Italian study (16) that showed that patients with HLA-loss at relapse frequently had active disease before allo-HSCT. Patients transplanted with a sizable leukemia burden probably present higher intratumoral heterogeneity and are therefore more likely to carry a clone with HLA-loss or with a predisposition to aUPD. In addition, a greater number of treatment lines was correlated in our study with a higher incidence of HLA-loss relapse (66 vs. 18%), given that chemotherapy agents might lead to a higher aUPD risk. As for pre-relapse cGVHD, 16% of the patients with HLA-loss had experienced cGVHD compared with 39% in the Crucitti et al. (16) study. In addition to the low number of patients with HLA-loss relapse in our study, the different GVHD prophylaxis employed [CsA/MTX and Sirolimus/MMF in Italian study (16) vs. Cy-post in our study] could explain the results for cGVHD as a risk factor.

HLA-loss relapses occur later after transplantation than classical relapses, a finding that has also been reported in other studies (16). This finding suggests that HLA-loss relapses might arise from *de novo* mutations occurring after Haplo-HSCT after a long phase of immune equilibrium between the donor's immune system and the residual leukemic cells. Pre-existing mutant clones would overcome the immune system more rapidly, and a classical relapse would occur earlier. Of note, this observation suggests that the post-transplantation follow-up of patients should be maintained over time.

Rescue treatment options after relapse post-Haplo-HSCT with PTCy aimed at improving the GVL effect (such as DLIs and immunosuppression withdrawal) could be a good option when patients present a lower disease burden (12, 13, 31, 32). However, such treatments would be ineffective in cases of HLA-loss relapses (33). In our study, three patients (1, 3, and 4) were treated with DLIs after HLA-loss relapse (analyzed retrospectively) and presented an initial response but subsequently had disease progression and died (median OS, was 375 days). Due to HLA-loss the donor T cells cannot recognize and kill leukemic cells. Moreover, the three patients presented severe GVHD after being administered DLIs, which required several lines of treatment including steroids and photopheresis in two patients. These patients were therefore not treated properly because the molecular mechanism underlying the relapse was unknown at the time the rescue treatment was selected. The two patients in which HLA-loss relapse was diagnosed prospectively were treated with alternative treatments not based on enhancing the GVL effect. One patient was administered brentuximab, a second Haplo-HSCT and nivolumab from a different donor and is still alive, while the other patient was administered daratumumab, mercaptopurine plus methotrexate and venetoclax but died from disease progression (Figure 1, Table 2, and Supplementary Table 2).

In contrast, DLIs were a better option for the patients with classical relapses, who presented a median OS of 750 days compared with the 375 days for the patients with HLA-loss relapse. Although treatment with DLIs at relapse can be a good option for this type of patient, the risks and benefits including the probability of developing GVHD should always be individualized for each patient.

Unfortunately, rescue treatments for post-transplantation relapse are far from effective, and only a minority of patients can be rescued in the long run. A better understanding of the relapse mechanisms will help improve survival and manage the complications associated with each treatment. The mechanisms by which leukemic cells evade immune control and lead to leukemia clonal evolution relapse after transplantation need to be studied (14). Taking into account this information will help to approach a personalized medicine management process and achieve long-term responses.

The main limitation of this study is its sample size, which might explain the reported observations that had no statistical significance. This limitation could be due to the fact that the study was conducted in a single center. In addition, the HLA-KMR quantitative PCR kit employed for the study only contains qPCR assays for HLA-A, HLA-C and HLA-DPB1 alleles. We could not therefore study all relapsed patients after haplo-HSCT. Further research is necessary with larger studies to obtain conclusive evidence of the reported observations.

In summary, these results confirm that HLA-loss relapses are a frequent event after haplo-HSCT with PTCy. This LOH phenomenon is an immune evasion mechanism that can confer a selective advantage to leukemic cells, which can thereby escape from donor immunosurveillance and lead to relapse. To design effective rescue strategies, the analysis of this immune evasion mechanism should be implemented in the routine management of patients who have undergone transplantation from haploidentical donors. Rescue treatments should not be based on DLIs or second transplantation with the same donor given that the GVL would not be effective in this setting.

Based on our results, in the routine management of patients transplanted from Haplo-HSCT or mismatched related donor we carry out chimerism analysis for post-transplantation follow-up. When we detected mixed chimerism, we confirm that patient has relapsed by morphology, flow cytometry, immunohistochemical, specific tumor marker or cytogenetics analysis. The recipient-specific HLA is selected according to the pre-HSCT HLA compatibility study among recipient and donor. If we can study recipient-specific HLA allele with HLA-KMR kit we carry out qPCR assays for HLA-loss study. The analysis of this immune evasion mechanism allow us to design effective rescue strategies.

## Data Availability Statement

The original contributions presented in the study are included in the article/Supplementary Material, further inquiries can be directed to the corresponding author/s.

## Ethics Statement

The studies involving human participants were reviewed and approved by the Ethics Committee of the Gregorio Marañón General University Hospital. The patients/participants provided their written informed consent to participate in this study.

## Author Contributions

CM-L and IB: funding acquisition, project administration, and supervision. PM, CM-L, and IB: formal analysis, visualization, and writing–original draft. PM, MK, JD-M, CM-L, and IB: conceptualization. PM and CM-L: data curation. PM, CM-L, and IB: formal analysis. PM, DC, MC, and CA-Z: methodology. MK, JD-M, CM-L, and IB: resources. PM, MK, CM-L, and IB: validation. PM, MK, DC, MC, RB, GO, JS-G, CA-Z, JM, ND, IG-C, JA, JD-M, CM-L, and IB: investigation and writing–review & editing. All authors contributed to the article and approved the submitted version.

## Conflict of Interest

The authors declare that the research was conducted in the absence of any commercial or financial relationships that could be construed as a potential conflict of interest.
