# Supplementary material for: Clinical Utility of the Detection of the Loss of the Mismatched HLA in Relapsed Hematological Patients After Haploidentical Stem Cell Transplantation With High-Dose Cyclophosphamide
Source: Front Immunol. 2021 Mar 25;12:642087. doi: 10.3389/fimmu.2021.642087 (PMC8027082; doi:10.3389/fimmu.2021.642087)
Supplement: Supplementary file 2 [file Data_Sheet_1.PDF]

Supplementary Figure 1. Overall survival of HLA loss and classical relapses. Kaplan Meier estimates for overall survival since relapse after transplantation in patients with HLA loss (red line) or classical (blue line) relapses.

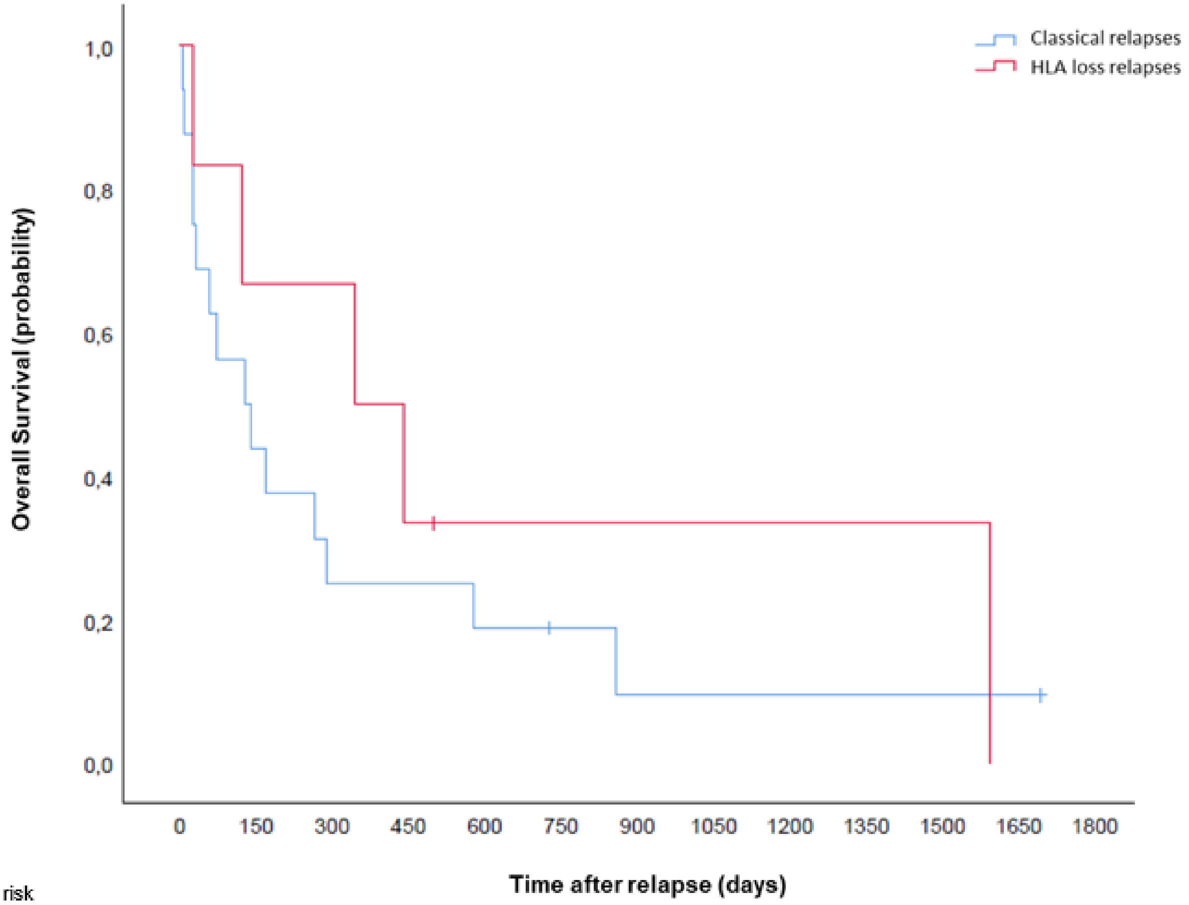

No. at risk													
HLA loss relapses	6	4	2	2	2	2	2	2	2	2	2	1	1
Classical relapses	16	7	4	4	3	3	2	2	2	2	2	2	2
