# Supplementary material for: Clinical Utility of the Detection of the Loss of the Mismatched HLA in Relapsed Hematological Patients After Haploidentical Stem Cell Transplantation With High-Dose Cyclophosphamide
Source: Front Immunol. 2021 Mar 25;12:642087. doi: 10.3389/fimmu.2021.642087 (PMC8027082; doi:10.3389/fimmu.2021.642087)
Supplement: Supplementary file 3 [file Data_Sheet_2.PDF]

Supplementary table 1. List of patient specific HLA (not shared by the donor) present in the cohort of 37 relapsed patients and identification of those used for analysis at relapse from included in the HLA detection kit used (HLA-KMR kit). NA: not available.

Patient	Patient specific HLA not shared by the donor	Patient HLA selected for study at relapse
1	A*68;C*08	A*68
2	C*04;A*03	C*04
3	A*01;C*06	A*01
4	C*03;A*24	C*03
5	C*04	C*04
6	C*04;A*03	C*04
7	A*68; C*17	A*68
8	A*24	A*24
9	A*11;C*05	A*11
10	A*01	A*01
11	C*04	C*04
12	A*26;C*12	A*26
13	A*02;C*05	A*02
14	A*11;C*01	A*11
15	A*68;C*04	A*68
16	A*02	A*02
17	A*01;C*06	A*01
18	A*24;C*04	A*24
19	A*25;C*06	A*25
20	A*02;C*04	A*02
21	A*02	A*02
22	A*02	A*02
23	A*03	NA
24	A*30, C*12	NA
25	A*23	NA
26	A*33, C*08	NA
27	C*06	NA
28	A*33, C*07	NA
29	A*03, C*14	NA
30	C*15,B*52	NA
31	A*23	NA
32	C*15	NA
33	C*12	NA
34	C*05	NA
35	A*03	NA
36	C*08	NA
37	A*23	NA
